# A New-Fangled *FES-k*-Means Clustering Algorithm for Disease Discovery and Visual Analytics

**DOI:** 10.1155/2010/746021

**Published:** 2010-06-08

**Authors:** Tonny J Oyana

**Affiliations:** 1 GIS Research Laboratory for Geographic Medicine, Advanced Geospatial Analysis Laboratory, Department of Geography & Environmental Resources, Southern Illinois University, 1000 Faner Drive, MC 4514, Carbondale, IL 62901-4514, USA

## Abstract

The central purpose of this study is to further evaluate the quality of the performance of a new algorithm. The study provides additional evidence on this algorithm that was designed to increase the overall efficiency of the original *k*-means clustering technique—the Fast, Efficient, and Scalable *k*-means algorithm (*FES-k*-means). The *FES-k*-means algorithm uses a hybrid approach that comprises the *k-d* tree data structure that enhances the nearest neighbor query, the original *k*-means algorithm, and an adaptation rate proposed by Mashor. This algorithm was tested using two real datasets and one synthetic dataset. It was employed twice on all three datasets: once on data trained by the innovative MIL-SOM method and then on the actual untrained data in order to evaluate its competence. This two-step approach of data training prior to clustering provides a solid foundation for knowledge discovery and data mining, otherwise unclaimed by clustering methods alone. The benefits of this method are that it produces clusters similar to the original *k*-means method at a much faster rate as shown by runtime comparison data; and it provides efficient analysis of large geospatial data with implications for disease mechanism discovery. From a disease mechanism discovery perspective, it is hypothesized that the linear-like pattern of elevated blood lead levels discovered in the city of Chicago may be spatially linked to the city's water service lines.

## 1. Introduction

Clustering delineates operation for objects within a dataset having similar qualities into homogeneous groups [1]. It allows for the discovery of similarities and differences among patterns in order to derive useful conclusions about them [2]. Determining the structure or patterns within data is a significant component in classifying and visualizing, which allows for geospatial mining of high-volume datasets. While there are many clustering techniques that have been developed over the years (many of which have been improvements and others have been revisions), the most common and flexible clustering technique is the *k*-means clustering technique [3]. The primary function of the *k*-means algorithm is to partition data into *k*disjoint subgroups, and then the quality of these clusters is measured via different validation methods. The original *k*-means method, however, is reputable for being feeble in three major areas: () computationally expensive for large-scale datasets; () cluster initialization a priori; and () local minima search problem [4, 5].

The first report to resolve these concerns about the *k-*means clustering technique was published as a book chapter [6]. In this paper, we have analyzed three distinct datasets and also make additional improvements in the implementation of the algorithm. Postprocessing work on discovered clusters involved a detailed component of fieldwork for one of the experimental datasets revealing key implications for disease mechanism discovery. This paper is inspired by an increasing demand for better visual exploration and data mining tools that function efficiently in data-rich and computationally rich environments. Clustering techniques have played a significant role to advance knowledge derived from such environments. Besides, they have been applied to several different areas of study, including, but not limited to, gene expression data [7, 8], georeferencing of biomedical data to support disease informatics research [9, 10] in terms of exploratory data analysis, spatial data mining, and knowledge discovery [11–13].

## 2. Algorithm Description

### 2.1. The *k*-Means Clustering Method

Several algorithms are normally used to determine natural homogeneous groupings within a dataset. Of all the different forms of clustering, the improvements suggested in this study are for the unsupervised, partitioned learning algorithm of the *k*-means clustering method [3]. MacQueen [3] describes *k*-means as a process for partitioning an *N*-dimensional population into *k* sets on the basis of a sample. Research shows that, to date, *k*-means is the most widely used and simplest form of clustering [14–16]. The *k*-means algorithm is formally defined, for this study, as follows.

(1) Let *k* be the number of clusters and the input vectors defined as .

(2) Initialize the centers to *k* random locations in the data and calculate the mean center of each cluster,  (where *i* is the th cluster center). 

(3) Calculate the distance from the center of each cluster to each input vector, assign each input vector to the cluster where the distance between itself and  is minimal, recompute  for all clusters that have inherited a new input vector, and update each cluster center (if there are no changes within the cluster centers, discontinue recomputation).

(4) Repeat step  until all the data points are assigned to their optimal cluster centers. This ends the cluster updating procedure with *k* disjoint subsets.

The partitions are based on a within-class variance, which measures the dissimilarity between input vectors , and cluster representatives  using the squared Euclidean distance: (1)

where *N* and *k* are the number of data and the number of cluster centers, respectively, *x _n_* is the data sample belonging to center  [3, 7, 17–19]. 

The center of the *k*th cluster is chosen randomly and according to the number of clusters in the data [8], where *k* can be used to manipulate the shape as well as the number of clusters. According to Vesanto and Alhoniemi [19], the *k*-means algorithm prefers spherical clustering, which assigns data to shapes whether clusters exist in the data or not, making it necessary to validate the results of the clusters. This can cause a problem because if a cluster center lies outside of the data distribution, the cluster could possibly be left empty, reflecting a dead center, as identified by Mashor [18]. Another weakness of the algorithm is its inability to deal with clusters having significantly different sizes [2].

### 2.2. Davies-Bouldin Validity Index (DBI)

The Davies-Bouldin Index (DBI) is used to evaluate clustering quality of the *k*-means partitioning methods because DBI is ideal for indexing spherical clusters. Hence, the ideal DBI for optimal clustering strives to minimize the ratio of the average dispersions of two clusters, namely *C _i_* and *C _j_*, to the Euclidean distance between the two clusters, according to the following formula [7, 20],(2)

where *k* is the number of clusters, *e _i_* and *e _j_* are the average dispersion of *C _i_* and *C _j_*, respectively. *D _ij_* is the Euclidean distance between *C _i_* and *C _j_*. The average dispersion of each cluster and the Euclidean distance are calculated according to formulas (2) and (3), respectively [7], (3)(4)

where is the center of cluster  consisting of  points and *x* is the input vector. 

Although research tells us that one advantage of the *k*-means algorithm is that it is computationally simplistic [2], the direct application of the algorithm to large datasets can be computationally very expensive because this method requires time proportional to the product of number of data points and the number of clusters per iteration [17, 19]. Vesanto and Alhoniemi [19] also suggested that DBI prefers compact scattered data. Unfortunately, not all data are compact and scattered; hence, an improved algorithm is required to evaluate very large data sets. This declaration comes 30 years after that of MacQueen [3] who proclaimed that the *k*-means procedure is easily programmed and is computationally economical.

### 2.3. The *k*-d Tree Data Structure

According to Bentley [21] and Gaede and Günther [22], the *k-d* tree is one of the most prominent *d*-dimensional data structures. The structure of the *k*-*d* tree is a multidimensional binary search mechanism that represents a recursive subdivision of the data space into disjoint subspaces by means of *d*-1-dimensional hyperplanes [14, 22, 23]. Note that the root of such a tree represents all the patterns, while the children of the root represent subsets of the patterns completely contained in subspaces. The nodes at the lower levels represent smaller subspaces.

The two main properties of the *k-d* tree are that each splitting hyperplane has to contain at least one data point and that nonterminal nodes must have one or two descendants. These properties make the *k-d* tree data structure an attractive candidate for reducing the computationally expensive nature of*k*-means algorithm and providing a very good preliminary clustering of a dataset [4, 14, 15, 17]. Several of these studies have investigated the use and efficiency of the *k-d* tree in a *k*-means environment, and they have concluded that presenting clustered data using this data structure provides enormous computational advantages. Alsabti et al.'s [17] main principle was based on organizing vector patterns so that all closest patterns to a given prototype can be found efficiently. The method consists of initial prototypes that are randomly generated or drawn randomly from the dataset. There are two main strategies to realize Alsabti's principle: () consider that all the prototypes were potential candidates for the closest prototype at the root level; () obtain good pruning methods based on simple geometrical constraints. 

Alsabti et al. [17] pruning method was based on computing the minimum and maximum distances to each cell. For each candidate , they obtained the minimum and maximum distances to any point in the subspace; then they found the minimum of maximum distances (MinMax); and later they pruned out all candidates with minimum distance greater than MinMax. For their pruning technique, Pelleg and Moore [23] used the bisecting hyperplane that assigns the input vector based on the minimal distance to the winning cell. Kanungo et al. [15] used the same approach, but they assigned the input vector to a cell based on minimal distance to the midpoint of the winning cell candidate. In this study, we have adopted the pruning method of Kanungo et al. [15] due to its presumed greater efficiency than that of Alsabti et al. [17] and Pelleg and Moore [23].

### 2.4. Mashor's Updating Method

A method intended to resolve the *k*-means problem has been described by Mashor [18], who suggested a multilevel approach. According to Vesanto and Alhoniemi [19], the primary benefit of a multilevel approach is the reduction of the computational cost. Recall that most clustering algorithms employ a similarity measure with a traditional Euclidean distance that calculates the cluster center by finding the minimum distance calculated using(5)

where *k* is the number of cluster centers, *N*is the total number of data points,  is the *n*th data point, and  is the *i*th cluster center. In *k*-means clustering as the data sample is presented, the Euclidean distances between the data sample and all the centers are calculated, and the nearest center is updated according to (6)

where  indicates the nearest center to the data sample . The centers and the data are written in terms of time (*t*), where  represents the cluster center during the preceding clustering step, and is the adaptation rate. The adaptation rate, , can be selected in a number of ways. Conventional formulas for are a variable adaptive method introduced by MacQueen [3] and a constant adaptation rate and a square root method introduced by Darken and Moody [24]. These methods adjust the cluster centers at every instant by taking the cluster center at the previous step into consideration. Some of the problems associated with such adjustments are reviewed in Mashor [18], who suggests a better clustering performance based on a more suitable adaptation rate . According to Mashor [18], a good updating method is one that has a large clustering rate at the beginning and a small steady state value of the adaptation rate, (*t*), at the end of training time. 

Mashor [18] investigated five methods—three conventional updating methods and two proposed. For this study, we adopted one of two proposed methods introduced by Mashor [18] into the Fast, Efficient, and Scalable *k*-means algorithm (*FES-k*-means algorithm). By intervening with the updating method, it is possible to facilitate the optimal cluster centers in gaining a good cluster performance.

### 2.5. FES-*k*-Means Algorithm

The purpose of this study is to address the problem that the *k*-means algorithm encounters while dealing with data-rich and computationally rich environments. Proposed modifications to produce the new algorithm, *FES-k*-means, begin by initializing the *k-d* tree data structure (based on a binary search tree that represents recursive subdivision) and using an efficient search mechanism based on the nearest neighbor query. This is expected to handle large geospatial data, reduce the computationally expensive nature of the *k*-means algorithm, and perform fast searches and retrieval. The next modification is to implement a more efficient updating method using Mashor's adaptation rate. The purpose of this step is to intervene at the updating stage of the *k*-means algorithm, because it suitably adjusts itself at each learning step in order to find the winning cluster for each data point efficiently, and it takes time into consideration and analyzes the cluster centers during the previous clustering steps while generating new cluster centers.

The three specific issues that will be addressed by implementing the proposed improvements of the *k*-means algorithm are as follows.

(1) From ongoing experimentation of using the *k*-means algorithm, it has been observed that the number of clusters fluctuate between 2+ and 2−. It is believed that Mashor's method stabilizes the number of clusters and converges faster.

(2) Vesanto and Alhoniemi [19] stated that DBI favors small number of clusters. Hence, the DBI will not serve a population of data with a very large number of clusters. It is assumed that the *k-d* tree in combination with Mashor's method will eliminate this problem also.

(3) Knowing that data clusters range in size and density, it is safe to say that Vesanto and Alhoniemi's [19] suggestion that because DBI prefers compact scattered data, it does not efficiently service all datasets. For instance, the spatial patterns or multidimensional nature of georeferenced data may not completely fit into the compact scattered data description. By intervening at the updating level, we expect Mashor's method to service the general population of datasets by eliminating this problem.

In *k*-means clustering an adaptive method is employed where the cluster centers are calculated and updated using (6). The plan of this study is to integrate Mashor's updating procedure, , in (7) into (6) to derive the most appropriate cluster centers,(7)

where . At each step of the learning, the adaptation rate should be decreased so that the weights of the training data can converge properly.

Formula (6) is rewritten by substituting  from formula (7) to obtain the final formula (8) as follows:(8)

It is hypothesized that the application of this updating procedure in (8) to the existing cost equation of the *k*-means will help generate clear and consistent clusters in the data. It is also assumed that the improved *k*-means algorithm if used in conjunction with the MIL-SOM algorithm [25] will provide a better result than the original *k*-means algorithm, which delineates cluster boundaries based on the best DBI validation. The MIL-SOM algorithm is essentially an improved version of the Self-Organizing Map (SOM), an unsupervised neural network that is used to visualize high-dimensional data by projecting it onto lower dimensions by selecting neurons or functional centroids to represent a group of valuable data [26].

Algorithm 1gives the pseudo code of the *FES-k*-means algorithm. The pseudo code for this hybrid approach primarily comprises the *k-d* tree data structure that enhances the nearest neighbor query, the original *k*-means algorithm, and an adaptation rate proposed by Mashor.

**Algorithm 1:**An improved pseudo code for the *FES-k*-means algorithm.

The basic structure of *FES-k* -means Algorithm

    () Determine the number and the dimensionality of points and set the number of clusters

         in the training set

    () Extract the data points

    () Construct a --tree for the data points in reference

    () Initialize centers randomly

    () Find closest points to the centers using nearest neighbor search

    () Find [center] as an array of centers of each cluster by centroid method

    () Choose an adaptation rate (eta) for -means with Mashor

    () **while** (max iterations reached)

         **for** each vector

         **for** each cluster

            Calculate the distance of vector to center of cluster

            Find the nearest cluster

         **end**

         Calculate eta = eta/exp(1/sqrt(cluster_count + iter))

         change_in_center = eta(difference between vector and cluster_center)

         Calculate new center = center + change_in_center

         **end**

         **if** (change_in_center)  epsilon

         break

         **end**

    // Compute MSE until it does not change significantly

    // Update centers until cluster membership no longer changes

    **end**

## 3. Materials and Methods

### 3.1. Experimental Design

**Figure 1:**
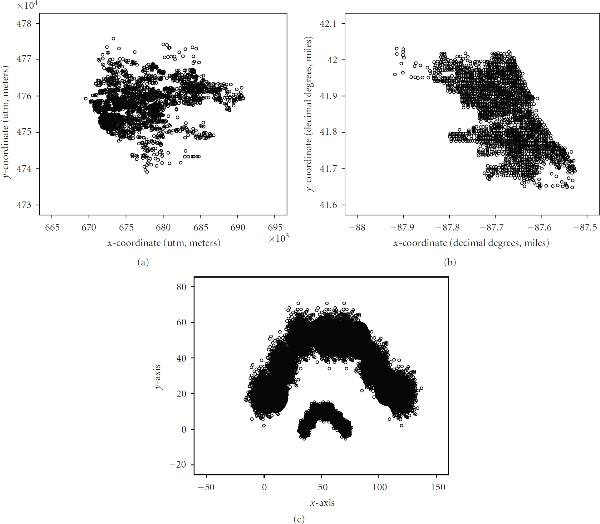
**The spatial distribution of the actual, untrained datasets: (a) adult asthma; (b) elevated blood lead levels linked with age of housing units; and (c) synthetic data**.

In this paper, we evaluated the characteristics and assessed the quality and efficiency of the *FES-k*-means clustering method. We invoked three distinct datasets to realize this goal. Two published real datasets and one published synthetic dataset were used for performance evaluation of the method. The data distribution is illustrated in Figure 1. The real datasets were () georeferenced physician-diagnosed adult asthma data for Buffalo, New York (Figure 1(a)); and () georeferenced elevated blood lead levels (BLLs) linked with the age of housing units in Chicago, Illinois (Figure 1(b)). Each of these datasets, that is the raw data in its entirety (untrained) and the reduced MIL-SOM trained version in conjunction with *FES-k*-means algorithm, was explored. The third, shown in Figure 1(c), is a computer-generated synthetic dataset with a predetermined number of clusters. Post processing work involved a detailed fieldwork on the BLL outliers generated after classification. Photographs were taken and collected evidence led to the development of superior study hypothesis.

### 3.2. Adult Asthma in Buffalo, New York

This dataset has variables depicting residential locations of adults with asthma in relation to pollution sites in Buffalo, New York, which were collected at individual level. The untrained set for these data comprises 4,910 records and the trained set contains 252 records. Both sets have 5 characterizing components: namely, geographic location based on - and -coordinates, case control code, distance to major road, distance to known pollution source, and distance to field-measured particulate matter. The last three variables were tracked using binary digits (0 and 1), where 1 indicates whether the given location is within 1,000 meters of the noted risk element and 0 otherwise.

### 3.3. Elevated BLL Linked with Age of Housing Units, Chicago Illinois

This dataset contained the age of housing units linked with the prevalence of children having elevated BLL in Chicago, Illinois. According to the US Centers for Disease Control and Prevention (CDC), elevated BLL has been formalized as all test results 10 g/dL (micrograms per deciliter). The untrained and trained datasets comprise 2,605 records and 260 records, respectively. These data are at census block group level. Both, the trained and untrained sets have the following 16 dimensions: (dimension 1) child population; (dimensions 2–10) homes built per decade, spanning pre-1935 to 1999; (dimension 11) median year of homes built; (dimension 12) elevated BLL prevalence in year 1997; (dimension 13) elevated BLL prevalence in year 2000; (dimension 14) elevated BLL prevalence in year 2003; and finally, (dimensions 15 and 16) geographic location based on - and -coordinates.

### 3.4. Synthetic Dataset

The published synthetic dataset (in 2-dimensional feature space,  = 36,000 data points with more than 10 clusters, all connected at the edges) was randomly generated. The untrained and trained dataset comprised 36,000 and 258 records, respectively. A pair of -, - coordinates was used to quantify its clusters.

### 3.5. Data Analysis

To achieve the goals of this research, we ran several tests employing the new *FES-k*-means clustering method. Our testing procedure comprised 3 major steps: () data preprocessing, () experimentation, and () data post processing. These experiments were conducted within improved MIL-SOM and *FES*-*k*-means environments using Matlab 7.0 (The MathWorks, Inc., Natick, Massachusetts). We decided on these computational environments to perform the algorithms because the MIL-SOM algorithm and Matlab provide the necessary environments to compute complex equations. Exploratory analyses were conducted using Statistical Programs, and spatial analysis was conducted using ESRI ArcGIS 9.2 (ESRI, Inc., Redlands, California).

### 3.6. Data Pre-Processing

This pre-processing consisted of selecting viable datasets that would be used for testing and validation. We chose published datasets because their characteristics are well established and adequately known, but this algorithm (*FES-k*-means) was initially tested using up to 1 million records generated randomly by the computer. The next step involved preparing the experimental datasets for modeling. After pre-processing the three datasets, they were imported into the work space environment for experimentation. 

### 3.7. Experimentation

During experimentation, we assessed the performance of the *FES-k*-means algorithm by performing three tasks: () evaluate speed efficiency using runtime; () evaluate mean square error for processed data; and () train the data. We compared the *FES-k*-means method with the standard *k*-means and with MacQueens *k*-means methods. MacQueen's *k*-means method, as referenced herein, is one that uses predefined parameters [18].

Using runtime, in seconds, speed efficiency was measured against percentage of data processed for each of the three aforementioned clustering methods. The percentage of data processed was based on percentages that ranged from 10 to 100 and increased in 10 percent increments (10%, 20%, 30%, etc.)

To test clustering quality of the *FES-k*-means method, we graphically compared the mean square error (MSE) measured in decibels (dB) of each dataset with the percentage of data processed using the three methods. 

Prior to cluster delineation of each dataset using the *FES-k*-means method, the data were separately trained using MIL-SOM. MIL-SOM training was used to initialize *k*—the number of clusters. SOM, in a geographical context, is used to reduce multivariate spatially referenced data to discover homogeneous regions and to detect spatial patterns [27]. In SOM, a winning neuron is randomly selected to represent a subset of data, while preserving the topological relationships [26]. The algorithm continues until all data are assigned to a neuron. Assignments are based on similarity characteristics using distance as a determinant; hence, similar data are grouped together and dissimilar clusters are assigned to separate clusters. The resulting clusters may be visualized using a multitude of techniques such as the *U*-matrix, histograms, and scatter plots, among others available within the SOM toolbox. For the purposes of our testing, we employed the *U*-matrix, which shows distances between neighboring units and displays cluster structure of the data. Clusters are typically uniform areas of low values; high values allude to large distances between neighboring map units and thus indicate cluster borders. 

For the trained version of each dataset, we initialized the number of centers, *k*, to 10; which proved to be insignificant in determining the number of major clusters. On the other hand, the initialized centers for the untrained data were varied; the BLL housing data had 6 centers; the adult asthma data was initialized to 8 clusters; and the synthetic dataset was initialized to 10 clusters. For each cluster center, 20 iterations were run. The number of clusters was estimated via visual interpretation of the *U*-matrix during the MIL-SOM training.

### 3.8. Data Post Processing

For post processing and validation, we complemented our *FES-k*-means with the traditional *k*-means algorithm in the SPSS and found that our method is comparable. Next, we wished to analyze cluster distribution, thus a box plot was undertaken. In a box plot, each record is plotted within a series of box plots corresponding to relative cluster groupings. We refer to these clusters as major "best as shown in the plots". Each case is graphed, within its cluster, based on distance from its classification cluster center. Visual probing and spatial analysis using box plots revealed hidden outliers, which prompted further investigation into the data. 

Next, we mapped the clusters and outliers using GIS to visualize, compare, and evaluate the cluster patterns and point distributions for the MIL-SOM trained sets and the full versions for each dataset. To further explore clusters and outliers, we did fieldwork and communal/housing investigations in Chicago, Illinois. Photos taken during this fieldwork are provided to support findings in relation to the link between BLL and potential risk factors.

## 4. Results

**Figure 2:**
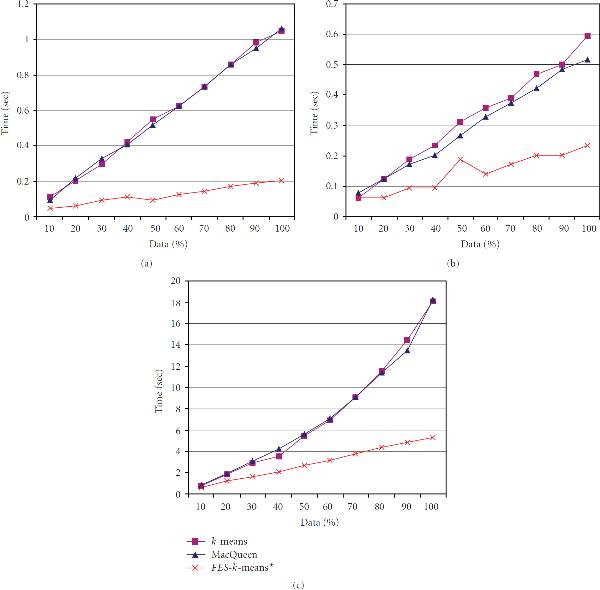
**A comparison of three -means algorithms using runtime versus percent of data processed: (a) adult asthma; (b) elevated blood lead levels linked with age of housing units; (c) synthetic data**.

**Figure 3:**
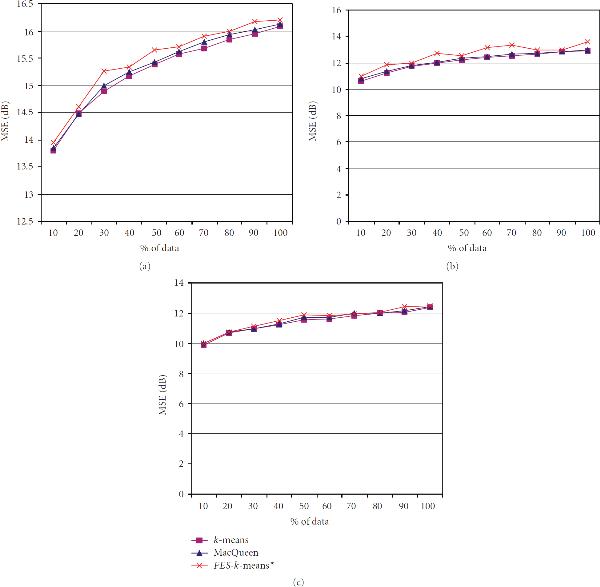
**A comparison of three -means algorithms using MSE versus percent of data processed: (a) adult asthma; (b) elevated blood lead levels linked with age of housing units; (c) synthetic data**.

**Figure 4:**
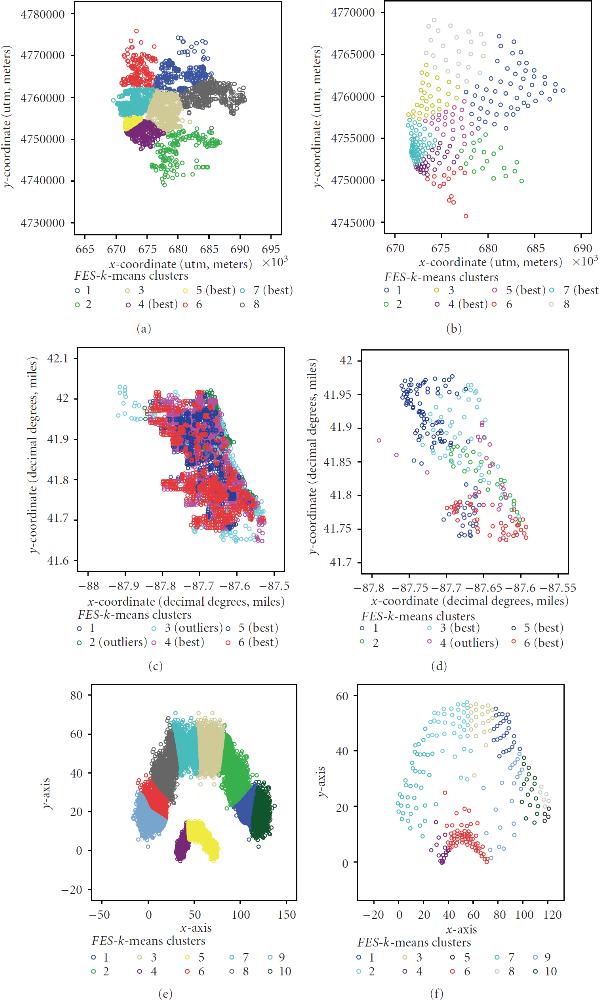
***FES-k*-means delineated boundaries of untrained and MIL-SOM trained data for: (a,b) adult asthma; (c,d) elevated blood lead levels linked with age of housing units; and (e,f) synthetic data**. (a,c,e) panel is the representation of untrained data, while on (b,d,f) is the representation of trained data.

**Figure 5:**
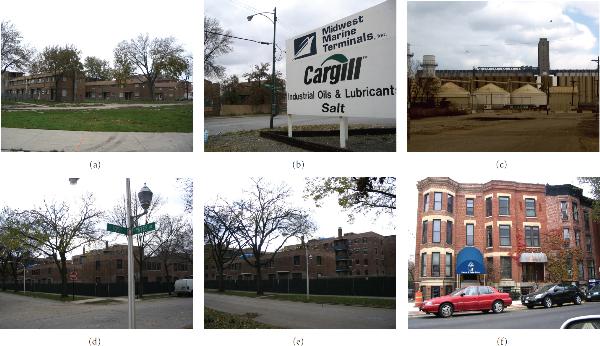
**Photos depicting housing conditions in Chicago, Illinois (taken in November 2006)**. The first five photos (Figures 5(a) through 5(e)) were taken in the west and southern part of city; and the last photo (Figure 5(f)) was taken in the northern part of the city. The fieldwork was partly based on the need to evaluate the quality of clusters and outliers identified in each class using the box plot while post processing. Figure 5(b) displays the locations where the photos were taken.

**Figure 6:**
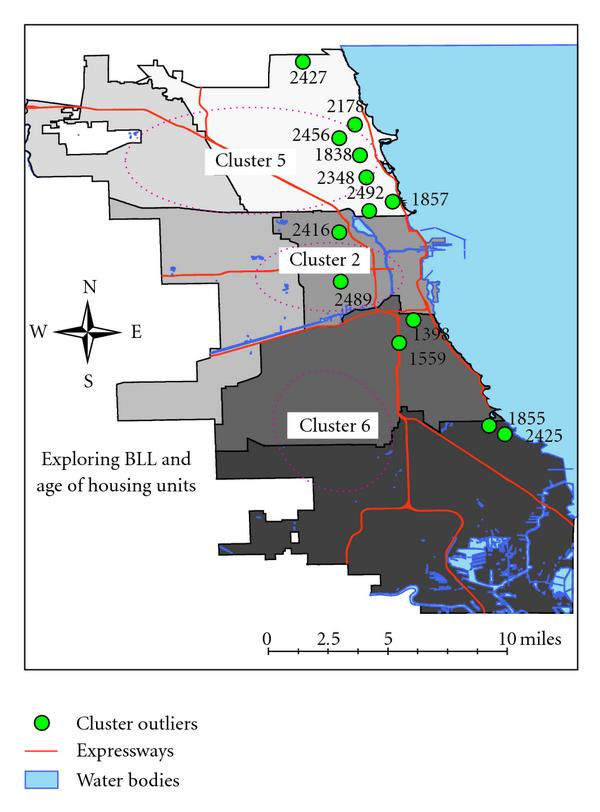
**Three delineated clusters in reference to age of housing units—revealing cluster outliers, proximity to major roadways, and water bodies in the City of Chicago**. We hypothesize that this linear-like pattern of elevated BLL may be spatially linked to the city's water service lines. This hypothesis begs a question: In the Chicago region, could lead pipes be a primary transportation medium for lead-contaminated water supply in schools, homes, and so forth?

Each dataset was evaluated using the *FES-k*-means algorithm to establish its key properties. Major benefits established during the implementation and experimentation were () it produces similar clusters as the original *k*-means method at a much faster rate; and () it allows efficient analysis of large geospatial data. The results identifying some of these main properties are presented in Figures 2 through 4. The first sets of illustrations (Figures 2 and 3) show the runtime and MSE results. The last illustration in Figure 4 shows delineated clusters of untrained and trained data. A key health outcome finding was deduced from the results of a postanalysis by the means of descriptive statistics, box plots, cluster quality re-evaluation using Davies-Bouldin validity index, and GIS analysis and fieldwork photos (Figures 5 and 6). 

### 4.1. Runtime

Figure 2(a) plots the runtime of the adult asthma dataset. The plot reveals that all three methods have a consistent, upward trend. For the standard *k*-means and MacQueen's methods, at 10 percent of the data processed, the runtime was 0.2 second, and at 100 percent, the runtime was just above 1 second. The runtime for the *FES-k*-means method was below 0.2 second for 10 percent of the data, but it remained at approximately 0.2 second for processing the remaining 90 percent of the data—a difference of at least 0.8 second from the other methods. 

The runtime for the elevated BLL dataset is displayed in Figure 2(b). The standard *k*-means, according to this plot, has the slowest runtime for the entire data processing; differing by no more than 0.8 second from MacQueen's method. Initially, the *FES-k*-means, at 10 percent of data processed, is analogous to that of the other methods. However, as the percentage of processed data increases, the runtime for the *FES-k*-means becomes increasingly faster, terminating at less than 0.25 second for 100 percent of the data. The end times for the standard *k*-means and the MacQueen's methods were approximately 0.6 second and 0.5 second, respectively. 

Figure 2(c) displays the runtime for the synthetic dataset. It is apparent that there is similarity in behaviors for all three methods, beginning at less than 1 second for 10 percent of data processed. As percentage of data increases, the runtime increases as well. The runtime for the standard *k*-means and MacQueen's methods increased greatly, while the time for *FES-k*-means increased only slightly. At 50 percent, for both the standard *k*-means and MacQueen's methods, the times were greater than 5 seconds, while it was less than 3 seconds for the *FES-k*-means; and the end runtimes, at 100 percent of data, were the same for the standard and MacQueen's at approximately 18 seconds, and approximately 6 seconds for the *FES-k*-means at the shortest time.

### 4.2. Mean Square Error

Figure 3 displays curves of the cluster performance of the standard *k*-means, the MacQueen method, and *FES-k*-means using MSE versus percentage of data processed. The Figure 3(a) curve reveals that all three methods have a consistent, increasing trend. The mean square error at the start of processing, 10 percent of data, is comparable for all methods at approximately 14 dB, and maximize, at 100 percent of data, slightly greater than 16 dB for each of the three methods. 

Figure 3(b) illustrates the elevated BLL block housing data. The characteristics of the standard *k*-means and MacQueen's methods, according to this plot, are very similar. Starting at an MSE of 11 dB for the standard -means, the MacQueen method, and the *FES-k*-means method and ending at an MSE of approximately 13 dB, the results indicate that the cluster performances are significantly close. 

In Figure 3(c), synthetic dataset, the cluster performance is comparable for all three methods: standard -means, MacQueen, and *FES-k*-means. The MSE at 10 percent of the data is 10, and it increases incrementally for each step of processing. At 100 percent of the data, the individual methods maximizes at an MSE slightly higher than 12 dB. The figure illustrates a continual increase in MSE with respect to percentage of data. 

### 4.3. FES-*k*-Means Clusters of MIL-SOM Trained versus Untrained Data

Both the MIL-SOM trained and untrained adult asthma datasets show similar geographic characteristics when the *FES-k*-means method is applied (Figures 4(a) and 4(b)). For the trained data, the spatial distribution for each of the clusters is more scattered than is the spatial distribution for the clusters of the actual data. Using less data points for the trained data may have caused this widespread spatial distribution of points in order to fully represent the data clusters of the actual data. The point pattern within this cluster is compact in the farthest south western portion of the cluster and is highly dense and compact. Also, as the cluster migrates northeast, it becomes more scattered and less compact and less dense.

Figures 4(c) and 4(d) illustrate the clustering results of untrained and MIL-SOM trained elevated BLL data. In comparison with the MIL-SOM trained data, we found that both the trained and untrained datasets returned comparable major clusters. The clusters for the MIL-SOM trained data capture clusters on the near west side and south side of Chicago; the untrained data reveal clusters in this same geographic area; in addition, a reference area was identified in the far north side. We also observe that the data points of the untrained data have a spatial distribution throughout the entire Chicago region (Figure 4(c)). This could be due in part to variations of noise presence within the data, not to mention that the untrained data are massively larger than the trained data by an approximate multiple of 10. Also, clusters 2 and 3 contain most of the outliers, which were explored further in a separate analysis and field study leading to the development of a study hypothesis. Overall, the *FES-k*-means clustering employed on MIL-SOM trained data and untrained data displays similar clustering characteristics for elevated levels of BLL with regards to the age of housing units for the city of Chicago.

Since we observed that the untrained elevated BLL linked with the age of housing dataset had two clusters with several outliers (Figure 4), we became curious about them. When these outliers were mapped, we found that most of them are primarily around the city perimeter and are within a distance of 1.50 miles from Lake Michigan. Prevalence rates within a 2-mile buffer radius of these outliers were analyzed using proximity and statistical analysis. The buffered areas only had the highest prevalence rate for all the three years under consideration, but also had the oldest housing units. Cluster outliers were further evaluated through a detailed fieldwork. 

Photographs taken as result of the fieldwork are provided in Figure 5. The photos were taken in November 2006 in different geographic areas within the identified clusters in the city of Chicago. Also, selected photos of housing units located in areas that reportedly had outliers are also included. For examples, outlier 2489 (sample photos were taken to show these outliers) is from Roosevelt Road to Laflin Street (Figure 5(a)) in the Chicago Housing Authority, it is also less than 1.5 miles along Lake Shore Drive. The housing units in this area are in the process of being demolished. Most units are vacant, though some residents still live there. Outlier 1398 is along 4000 South King Drive (Figure 5(e)). It is a lower middle class neighborhood and runs along Lake Shore Drive. Outlier 2492 is from Pulaski Road to Lawrence Avenue (Figure 5(f)) and is an upper class neighborhood. 

Three major clusters were identified in Figure 6: clusters 2 and 6 have elevated BLL, while Cluster 5 has the lowest BLL (this can be used as a reference in epidemiological investigations). Cluster 6, shown by two sample photos; is from 107th Street and Commercial Avenue (Figures 5(b) and 5(c)) to 105th Street and Yates Boulevard (Figure 5(d)); it includes the Industrial Belt and Cargill Industrial Plant and is near the Altgeld Gardens Housing Projects. Also, located in the same cluster is the Chicago Housing Authority where some of the units are being renovated. 

A significant number of outliers were observed in the southeast side, far north region of Chicago along its borderline and north suburb. *We hypothesize that this linear-like pattern of elevated BLL may be spatially linked to the city's water service lines. This hypothesis begs this question: in the Chicago region, could lead pipes be a primary transportation medium for lead-contaminated water supply in schools, homes, and so forth?*In reviewing the history of the city with regards to the water service lines and despite the fact that the ban on lead service mains was effected in 1988—critical information contained in 1993 Consumer Reports and also in Wald, M.L., May 12, 1993, The New York Times—we discovered that Chicago had lead levels which had more than 15 parts per billion in the 17 percent of the first draw samples.

Regarding pediatric lead exposure, the overall prevalence rates for 1997, 2000, and 2003 continuously declined as the years passed. We also found that the prevalence rates were higher in areas with older housing units. Lastly, we observed higher prevalence rates in areas with high minority presence and lower prevalence rates in areas with low minority presence. The reference area identified in previous studies, the northernmost region, is analogous to the findings in this study. The *FES-k*-means was efficient in discovering a cluster within a cluster, which was otherwise unnoticed in previous studies. *Findings from this study therefore prompt investigation of soil samples to investigate whether there is an association between potential water contamination in water service lines and elevated BLL presence. Another study would be to sample school children from all Chicago neighborhoods to investigate any effects lead poisoning may have on their learning abilities despite children's socioeconomic status.*

### 4.4. FES-*k*-Means Clusters of Synthetic Dataset

Figures 4(e) and 4(f) give the plot of the delineated synthetic dataset. We identified 10 clusters. The clusters closest to the origin are more concentrated than those that are farther away from the origin. In other words, as the - and -coordinates increase, the clusters become less dense in Figure 4(f). In Figure 4(e), the clusters of the untrained data are compact and highly dense. The formed clusters are primarily well defined and distinguished. This figure clearly shows that the 10 clusters found in here are a good representation of the clusters of the original data. Both figures show that the clusters for the synthetic dataset using the *FES-k*-means clustering approach are comparable.

## 5. Discussions

In this study, we have presented an improved clustering algorithm that overcomes some of the problems commonly associated with the conventional *k*-means algorithm. Justification of our focusing on *k*-means is primarily because it is a standard technique, is commonly used in a variety of applications, is employed on different software platforms, and is fairly easy to manipulate. Our goal of this study was to further explore this newly developed algorithm and understand its capacity in terms of disease mechanism discovery. Based on the BLL dataset, we detected a robust and consistent pattern of elevated blood levels among children that was completely missed in previous analysis. This lead to the formulation of a new study hypothesis revealing that the *linear-like pattern of elevated BLL discovered in this analysis may be spatially linked to the city's water service lines.*

In terms of the benefits and properties of using this algorithm, previous studies like those of Alsabti et al. [17], Pelleg and Moore [23], and Kanungo et al. [15] used a two-level approach by employing the *k-d* tree data structure. Other integrative studies, those by Vesanto and Alhoniemi [19] and Yano and Kotani [8] used the approach of combining a self-organizing map and *k*-means clustering for analyzing data. In this study, we explored the new algorithm separately using untrained datasets and also applied it to MIL-SOM trained datasets. Its design and implementation involved the application of the *k-d* tree data structure, nearest neighbor query, and a modified method to perform cluster updating by employing Mashor's adaptation rate. To the best of our knowledge no study has synthesized a conglomerate of these methods to enhance the efficiency in the *k*-means algorithm. The major benefit of our method is its procedural complexity that provides better speed and its strength has been demonstrated on real-world spatial datasets and synthetic datasets.

Although other clustering algorithms and many derivatives of the *k*-means algorithm have been introduced in the literature, the *FES-k*-means method has the advantage of yielding efficient clusters when used in combination with the MIL-SOM algorithm. We used the *U*-matrix (Sammon's mapping may also be used) results from the MIL-SOM training data to determine the value of *k*. This then enabled us to establish initial parameters for the number of clusters in the data, foregoing our dependency on costly computational wrapper methods like *k*-means with random restart [14] or the costly task of searching for the best initializations possible used by Bradley and Fayyad [28], among many other researchers. 

Our research confirmed previous studies that the *training—clustering* combination provides considerably better clusters than clustering without training. We witnessed that clustering formed from the MIL-SOM trained data is very similar and acceptable to the clusters formed by clustering the data directly. Other studies suggest that using a two-stage data reduction technique significantly improves clustering over clustering the data directly [29]. This two-stage procedure of clustering prototype vectors reduces the computation time, enabling clustering of large geospatial data [19].

Let us consider a few examples to support our claims. Though limited in available literature, with the new -windows approach [5], the windows have to be predetermined and input by the user. The density-based clustering algorithm DBSCAN accounts for arbitrary shapes and varying cluster sizes [30]. However, it has two input parameters that are limiting, the noise percentage has to be determined by and input by the user; the minimum number of points is automatically preset to 4 for all two-dimensional datasets; and lastly it assumes uniform cluster density—all pointing to a very limited fate with real-life applications on large, high-dimensional datasets. CLARANS is another incompetent algorithm because it is prohibitive on large databases—when dealing with large clusters, it has the tendency to split large clusters, and it has no explicit notion of dealing with noise [31]. In addition, studies performed by Ester et al. [30] have reported that DBSCAN is superior to CLARANS. Therefore, though not scientifically proven, we can assume that if *FES-k*-means is superior to DBSCAN; it also outperforms CLARANS. The CURE (clustering using representatives) uses cluster representatives that are found using a shrinking method [32]. Although this method can find arbitrary shapes and cluster sizes, the algorithm can incorrectly merge clusters. The Chameleon method partitions data into subclusters and then repeatedly combines them to obtain final clusters [33]. This method also relies on user-specified thresholds for its input parameters, relative interconnectivity, and relative closeness between cluster pairs in order to correctly merge clusters, possibly resulting in under- or overestimates of interconnectivity. To date, it has not proven successful on data with more than two dimensions and does not accurately compute values for small clusters. Consequently, we can assume that *FES-k*-means outperforms each of these methods because the aforementioned problems are inherently addressed by the novel *FES-k*-means method. 

Standard *k*-means has a time complexity based on the product of the number of patterns, *N*, the number of clusters, *k*, and the number of iterations—overwhelmingly increasing costs for large datasets [34]. We discount this costly computation by reducing the number of patterns examined using the well-known *k-d* tree data structure. The implementation of the *k-d* tree structure is used in collaboration with nearest neighbor query to maximize indexing and to provide a well-organized search and retrieval mechanism. This approach introduces an efficient storage structure that reduces the computational cost of match queries and is so dynamic that it can be employed by many applications according to Bentley [21] and Likas et al. [14]. Data tree structures provide stability to the data structure as mentioned by Kanungo et al. [15], along with better partitioning accuracy [5] and preliminary clustering of the dataset [14]. There are two main approaches for the overall *k-d* structure: () splitting using the median-based approach or midpoint-based approach and () splitting across the dimensions or along the lengthiest side. We built the *k-d* tree structure according to the suggestion of Alsabti et al. [17] using the midpoint-based approach along the lengthiest side, which they claim is the best *k-d* pruning approach. Although more complex pruning strategies are available, we found Kanungo's pruning approach to be efficient and did not require much computation time [17]. 

*FES-k*-means was written in C and was then exported and utilized in Matlab in conjunction with the MIL-SOM algorithm. The efficiency and robustness of the algorithm enables it to be used on multiple platforms and program applications. The runtime plots for each dataset for each method report that the *FES-k*-means scales linearly with the percentage of data. It is important to note that the variation in the data amounts in these datasets ranges from 2,605 records to 36,000 records; some scattered others are tightly compact. These plots reveal that, unlike conventional *k*-means, *FES-k*-means is not as sensitive to the size and distribution of the data. Note that the untrained data is plagued with noise and outliers. Initializing the clusters using the MIL-SOM algorithm enables effective management of these outliers. Furthermore, post processing using box plots shows even greater performance of cluster formation and is instrumental in identifying cluster outliers immediately.

## 6. Conclusions and Future Directions

The *FES-k*-means algorithm uses a hybrid approach that comprises the *k-d* tree data structure [21], nearest neighbor query for the *k-d* tree [35], the original *k*-means algorithm [3], and an adaptation rate proposed by Mashor [18]. The main properties established during the implementation and experimentation with the *FES-k*-means algorithm is as follows: () it produces clusters similar to the original *k*-means method at a much *faster* rate; and () it provides efficient analysis of large geospatial data with implications for disease mechanism discovery. From a disease mechanism discovery perspective, it is hypothesized that the linear-like pattern of elevated blood lead levels discovered in the city of Chicago may be spatially linked to the city's water service lines.

Additional observations made in this study that further characterize the *FES-k*-means clustering approach are as follows: () clustering previously trained data using the MIL-SOM method is more beneficial than clustering an entire dataset; () knowledge can be discovered based on outlier detection that was otherwise undistinguishable by traditional methods; and () *FES-k-*means clustering algorithm produces interesting information that can lead to further discoveries.

Possible expansion of the *FES-k*-means algorithm may revolve around ways to evaluate and measure cluster and subcluster qualities of the *FES-k*-means method by use of established or newly developed dissimilarity calculations; use of *FES-k*-means on nonnumeric data; implementation of parallel processing for acceleration; and ability to handle even larger datasets simultaneously. We believe that error tracking at each major step of the algorithm will help to improve the overall mean square error. Also, *FES-k*-means has been developed to handle point data; therefore it is limited in its ability to cluster other data types—that is, lines or polygons. 

Future developments for clustering, in general, may include the ongoing effort on how to effectively visualize multidimensional data; and as with the case of all clustering algorithms, clusters formed via one cluster performance are not necessarily the same clusters formed on processes thereafter, focusing on an algorithm that can return identical cluster structures for each subsequent cluster procedure of a given dataset is a future attempt for cluster optimization.

## Protection of Human Subjects

All research was approved by the Southern Illinois University Carbondale Human Investigation Review Board in accordance with national and institutional guidelines for the protection of human subjects.
